# 4-Amidino­pyridinium hexa­chlorido­stannate(IV) dihydrate

**DOI:** 10.1107/S241431462200195X

**Published:** 2022-03-03

**Authors:** Rochdi Ghallab, Hassiba Bougueria, Hocine Merazig

**Affiliations:** a Environmental Molecular and Structural Chemistry Research Unit, University of Constantine-1, 25000, Constantine, Algeria; b Centre Universitaire Abdelhafid Boussouf - Mila, Algeria; University of Aberdeen, Scotland

**Keywords:** crystal structure, hexa­chlorido­stannate(IV), 4-amidino­pyridinium

## Abstract

In the title hydrated mol­ecular salt {systematic name: 4-[amino­(iminium­yl)meth­yl]pyridin-1-ium hexa­chlorido­stannate(IV) dihydrate}, (C_6_H_9_N_3_)[SnCl_6_]·2H_2_O, the tin atom lies on a crystallographic inversion centre and the organic cation shows whole-mol­ecule disorder. Numerous N—H⋯O, N—H⋯Cl and O—H⋯Cl hydrogen bonds link the components in the crystal.

## Abstract 2

The organic cation in the title compound shows whole-mol­ecule disorder.

## Graphical abstract

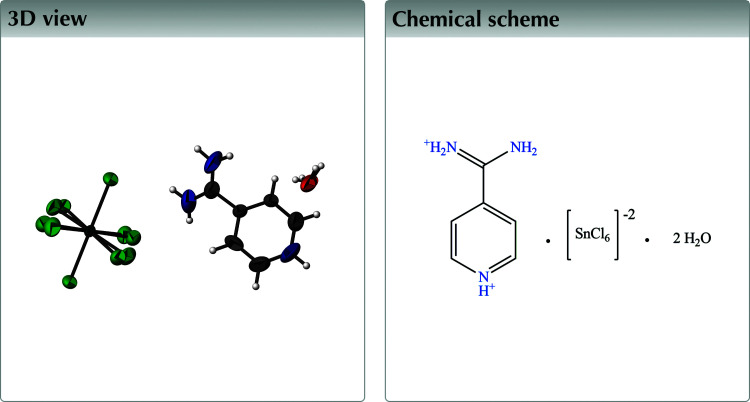

## Structure description

**Figure 1:**
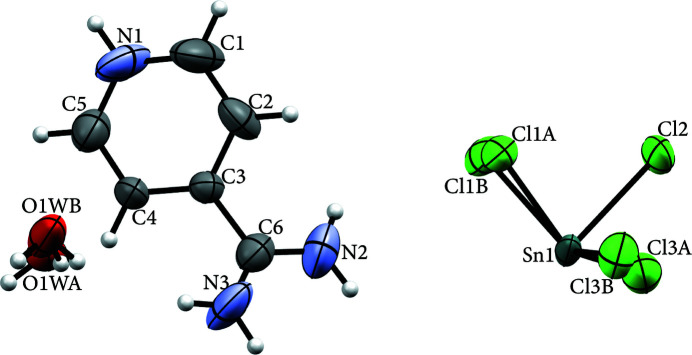
The mol­ecular structure showing 30% displacement ellipsoids.

The title hydrated mol­ecular salt, with formula (C_6_H_9_N_3_)·[SnCl_6_]·2H_2_O, crystallizes in the triclinic space group *P* 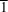 . The asymmetric unit is constituted by a Sn_0.5_Cl_3_ fragment (Sn site symmetry 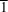 ), a 4-amidino­pyridinium cation (twice protonated at N1 and N2) and a water mol­ecule, as shown in Fig. 1 ▸.

The cation shows whole-mol­ecule disorder about an inversion centre and the water mol­ecule is disordered over adjacent positions (O⋯O = 1.13 Å) and there is also static disorder of two of the chloride ions of the anion. With the exception of Cl3, where the occupancy ratio is 0.67/0.33 (for Cl3*A*/Cl3*B*), each disordered atom is shared between two crystallographic sites with occupancies of 0.50. There are no abnormalities in the bond lengths and angles and they are comparable to those of similar types (Liu *et al.*, 2011 ▸; Ghallab *et al.*, 2020 ▸).

**Figure 2:**
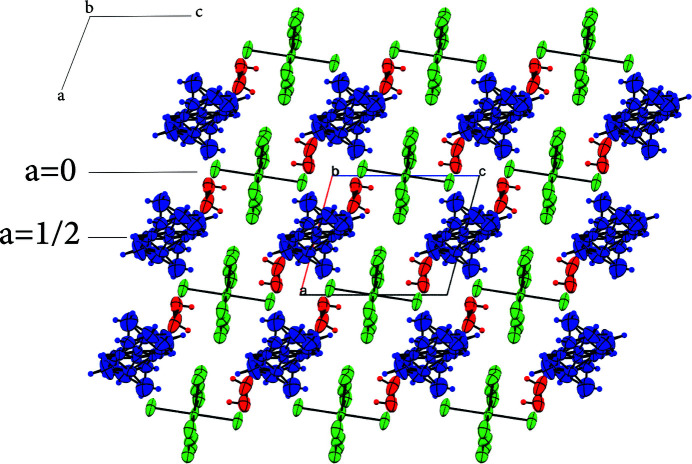
Projection of the crystal packing on the *ac* plane.

In the extended structure, cationic and anionic layers occur, with water mol­ecules inter­calating between them as shown in the projection of the structure onto the *ac* and *bc* planes (Figs. 2 ▸ and 3 ▸). Cohesion in the crystal is ensured by numerous hydrogen bonds (Table 1 ▸).

## Synthesis and crystallization

Following the method of preparation described in the literature (Bouchene *et al.*, 2018 ▸), the compound was synthesized *via* the aqueous technique. A millimeter-sized transparent crystal was formed after three months of slow evaporation at ambient temperature.

## Refinement

Crystal data, data collection and structure refinement details are summarized in Table 2 ▸. The disordered atoms were treated with constraints on distances and angles (by the SAME command and PART options). With the exception of Cl3, where the ratio is 0.67/0.33, each disordered atom is shared between two crystallographic sites with occupancy rates of 0.50.

## Supplementary Material

Crystal structure: contains datablock(s) I. DOI: 10.1107/S241431462200195X/hb4399sup1.cif

Structure factors: contains datablock(s) I. DOI: 10.1107/S241431462200195X/hb4399Isup2.hkl

CCDC reference: 2153109

Additional supporting information:  crystallographic information; 3D view; checkCIF report

## Figures and Tables

**Figure 3:**
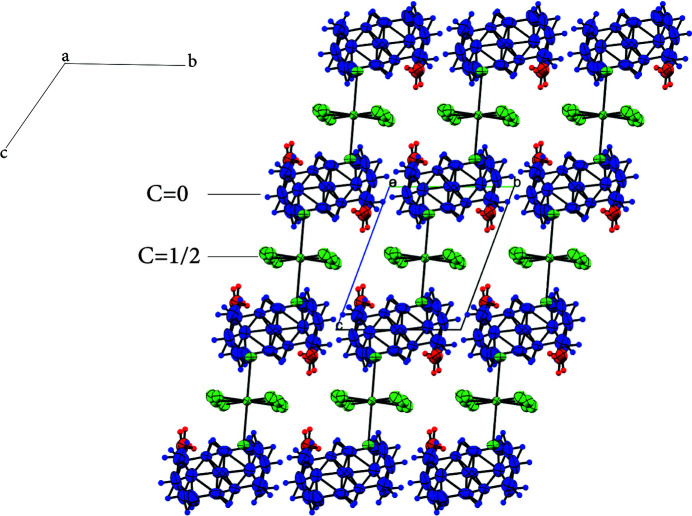
Projection of the crystal packing on the *bc* plane.

**Table 1:** Hydrogen-bond geometry (Å, °)

*D*—H⋯*A*	*D*—H	H⋯*A*	*D*⋯*A*	*D*—H⋯*A*
N1—H1⋯O1*WA* ^i^	0.86	1.96	2.760 (15)	154
N1—H1⋯O1*WB* ^i^	0.86	1.87	2.649 (15)	149
N2—H2*A*⋯Cl2^ii^	0.86	2.68	3.431 (11)	147
N3—H3*A*⋯O1*WA* ^iii^	0.86	2.13	2.961 (16)	162
N3—H3*A*⋯O1*WB* ^iii^	0.86	1.96	2.795 (16)	163
N3—H3*B*⋯Cl1*A* ^iv^	0.86	2.69	3.093 (16)	110
N3—H3*B*⋯Cl3*B* ^iv^	0.86	2.56	3.420 (16)	175
O1*WA*—H1*WA*⋯Cl2^v^	0.85	2.77	3.415 (8)	134
O1*WA*—H1*WB*⋯Cl3*A* ^vi^	0.85	2.41	3.154 (9)	147
O1*WB*—H1*WC*⋯Cl1*A* ^v^	0.85	2.60	3.305 (10)	142
O1*WB*—H1*WC*⋯Cl1*B* ^v^	0.85	2.36	3.085 (10)	144
O1*WB*—H1*WC*⋯Cl1*A* ^vii^	0.85	2.69	3.251 (12)	124
O1*WB*—H1*WC*⋯Cl1*B* ^vii^	0.85	2.83	3.396 (13)	126
C1—H1*A*⋯Cl3*A* ^viii^	0.93	2.67	3.561 (17)	161
C1—H1*A*⋯Cl3*B* ^viii^	0.93	2.43	3.356 (17)	174
C5—H5⋯Cl1*A* ^vii^	0.93	2.80	3.674 (12)	157
C5—H5⋯Cl1*B* ^vii^	0.93	2.56	3.385 (12)	149

Symmetry codes: (i) 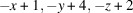 ; (ii) 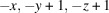 ; (iii) 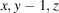 ; (iv) 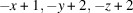 ; (v) 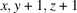 ; (vi) 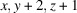 ; (vii) 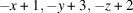 ; (viii) 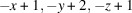 .

**Table 2:** Experimental details

Crystal data	
Chemical formula	(C_6_H_9_N_3_)[SnCl_6_]·2H_2_O
*M*_r_	490.58
Crystal system, space group	Triclinic, *P* 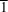
Temperature (K)	296
*a*, *b*, *c* (Å)	7.4224 (13), 7.4518 (11), 8.4986 (16)
α, β, γ (°)	105.726 (7), 97.426 (9), 112.383 (7)
*V* (Å^3^)	403.85 (12)
*Z*	1
Radiation type	Mo *K*α
μ (mm^−1^)	2.57
Crystal size (mm)	0.17 × 0.13 × 0.11

Data collection	
Diffractometer	Bruker APEXII CCD
Absorption correction	Multi-scan (*SADABS*; Bruker, 2016 ▸)
*T*_min_, *T*_max_	0.676, 0.754
No. of measured, independent and observed [*I* > 2σ(*I*)] reflections	10469, 2442, 1889
*R*_int_	0.028
(sin θ/λ)_max_ (Å^−1^)	0.714

Refinement	
*R*[*F* ^2^ > 2σ(*F* ^2^)], *wR*(*F* ^2^), *S*	0.046, 0.085, 1.15
No. of reflections	2442
No. of parameters	154
No. of restraints	53
H-atom treatment	H-atom parameters constrained
Δρ_max_, Δρ_min_ (e Å^−3^)	1.22, −1.35

Computer programs: *APEX2* and *SAINT* (Bruker, 2016 ▸), *olex2.solve* (Bourhis *et al.*, 2015 ▸), *SHELXL* (Sheldrick, 2015 ▸) and *OLEX2* (Dolomanov *et al.*, 2009 ▸).

## full crystallographic data

### Crystal data

**Table:**

(C_6_H_9_N_3_)[SnCl_6_]·2H_2_O	*Z* = 1
*M**_r_* = 490.58	*F*(000) = 238
Triclinic, *P*1	*D*_x_ = 2.017 Mg m^−^^3^
*a* = 7.4224 (13) Å	Mo *K*α radiation, λ = 0.71073 Å
*b* = 7.4518 (11) Å	Cell parameters from 1889 reflections
*c* = 8.4986 (16) Å	θ = 5.0–30.5°
α = 105.726 (7)°	µ = 2.57 mm^−^^1^
β = 97.426 (9)°	*T* = 296 K
γ = 112.383 (7)°	Block, colourless
*V* = 403.85 (12) Å^3^	0.17 × 0.13 × 0.11 mm

### Data collection

**Table:**

Bruker APEXII CCD diffractometer	1889 reflections with *I* > 2σ(*I*)
φ and ω scans	*R*_int_ = 0.028
Absorption correction: multi-scan (SADABS; Bruker, 2016)	θ_max_ = 30.5°, θ_min_ = 5.0°
*T*_min_ = 0.676, *T*_max_ = 0.754	*h* = −10→10
10469 measured reflections	*k* = −10→10
2442 independent reflections	*l* = −12→12

### Refinement

**Table:**

Refinement on *F*^2^	Primary atom site location: iterative
Least-squares matrix: full	Hydrogen site location: mixed
*R*[*F*^2^ > 2σ(*F*^2^)] = 0.046	H-atom parameters constrained
*wR*(*F*^2^) = 0.085	*w* = 1/[σ^2^(*F*_o_^2^) + (0.0167*P*)^2^ + 0.8036*P*] where *P* = (*F*_o_^2^ + 2*F*_c_^2^)/3
*S* = 1.15	(Δ/σ)_max_ < 0.001
2442 reflections	Δρ_max_ = 1.22 e Å^−^^3^
154 parameters	Δρ_min_ = −1.35 e Å^−^^3^
53 restraints	

### Special details

**Table:**

Geometry. All esds (except the esd in the dihedral angle between two l.s. planes) are estimated using the full covariance matrix. The cell esds are taken into account individually in the estimation of esds in distances, angles and torsion angles; correlations between esds in cell parameters are only used when they are defined by crystal symmetry. An approximate (isotropic) treatment of cell esds is used for estimating esds involving l.s. planes.

### Fractional atomic coordinates and isotropic or equivalent isotropic displacement parameters (Å^2^)

**Table:**

	*x*	*y*	*z*	*U*_iso_*/*U*_eq_	Occ. (<1)
Sn1	0.000000	0.500000	0.500000	0.04673 (15)	
Cl2	−0.0563 (2)	0.39683 (16)	0.19228 (11)	0.0675 (4)	
Cl1B	0.2964 (10)	0.7987 (8)	0.5355 (8)	0.0640 (13)	0.5
Cl3B	0.2133 (9)	0.3209 (8)	0.5250 (8)	0.0607 (14)	0.33
Cl1A	0.3472 (10)	0.7539 (8)	0.5316 (7)	0.0612 (12)	0.5
Cl3A	0.1294 (5)	0.2493 (4)	0.4986 (4)	0.0711 (9)	0.67
C3	0.470 (3)	1.475 (3)	0.998 (2)	0.039 (3)	0.5
C4	0.5060 (13)	1.6621 (11)	1.1133 (9)	0.0462 (19)	0.5
H4	0.465642	1.667299	1.212925	0.055*	0.5
C5	0.5998 (16)	1.8390 (14)	1.0820 (13)	0.062 (2)	0.5
H5	0.618436	1.965008	1.157590	0.074*	0.5
N1	0.666 (2)	1.8325 (16)	0.9421 (16)	0.081 (3)	0.5
H1	0.734961	1.946420	0.927815	0.097*	0.5
C1	0.625 (3)	1.6494 (18)	0.8222 (18)	0.088 (5)	0.5
H1A	0.661958	1.646634	0.721374	0.105*	0.5
C2	0.5291 (16)	1.4684 (15)	0.8517 (10)	0.059 (2)	0.5
H2	0.504299	1.342167	0.772395	0.071*	0.5
C6	0.3621 (14)	1.2750 (14)	1.0199 (13)	0.053 (2)	0.5
N2	0.227 (2)	1.1247 (14)	0.8868 (15)	0.099 (4)	0.5
H2A	0.153059	1.008127	0.895028	0.119*	0.5
H2B	0.211066	1.142421	0.790976	0.119*	0.5
N3	0.397 (2)	1.2666 (18)	1.1621 (18)	0.089 (5)	0.5
H3A	0.329233	1.154902	1.180199	0.106*	0.5
H3B	0.488756	1.372373	1.243683	0.106*	0.5
O1WA	0.0858 (13)	1.8847 (11)	1.1795 (10)	0.069 (2)	0.5
H1WA	0.119362	1.785736	1.167388	0.103*	0.5
H1WB	0.101422	1.945036	1.284078	0.103*	0.5
O1WB	0.2372 (18)	1.8798 (12)	1.1997 (9)	0.091 (3)	0.5
H1WC	0.297336	1.913349	1.302646	0.137*	0.5
H1WD	0.110355	1.828348	1.187657	0.137*	0.5

### Atomic displacement parameters (Å^2^)

**Table:**

	*U*^11^	*U*^22^	*U*^33^	*U*^12^	*U*^13^	*U*^23^
Sn1	0.0750 (3)	0.02256 (16)	0.02323 (16)	0.00466 (17)	0.00409 (16)	0.00756 (12)
Cl2	0.1091 (10)	0.0438 (5)	0.0264 (4)	0.0154 (6)	0.0077 (5)	0.0086 (4)
Cl1B	0.064 (3)	0.046 (2)	0.0547 (17)	−0.0007 (15)	−0.0018 (17)	0.0211 (17)
Cl3B	0.067 (4)	0.051 (3)	0.056 (2)	0.025 (2)	−0.004 (2)	0.019 (2)
Cl1A	0.072 (3)	0.0429 (19)	0.0492 (14)	0.0061 (14)	0.0161 (18)	0.0157 (13)
Cl3A	0.111 (3)	0.0488 (14)	0.0504 (13)	0.0290 (14)	0.0200 (16)	0.0222 (12)
C3	0.041 (10)	0.040 (8)	0.038 (3)	0.018 (7)	0.013 (5)	0.018 (4)
C4	0.057 (5)	0.041 (4)	0.033 (3)	0.014 (4)	0.012 (3)	0.013 (3)
C5	0.058 (6)	0.048 (5)	0.073 (6)	0.019 (5)	0.020 (5)	0.017 (4)
N1	0.096 (9)	0.065 (6)	0.116 (9)	0.038 (6)	0.054 (8)	0.064 (7)
C1	0.137 (12)	0.087 (9)	0.092 (9)	0.067 (10)	0.081 (8)	0.058 (8)
C2	0.083 (7)	0.075 (6)	0.039 (4)	0.053 (6)	0.023 (4)	0.017 (4)
C6	0.049 (6)	0.045 (4)	0.064 (6)	0.019 (4)	0.018 (5)	0.020 (4)
N2	0.120 (10)	0.042 (4)	0.094 (8)	0.009 (6)	0.017 (7)	0.005 (5)
N3	0.094 (9)	0.045 (6)	0.092 (9)	−0.005 (6)	−0.006 (7)	0.037 (6)
O1WA	0.094 (6)	0.049 (4)	0.053 (4)	0.014 (4)	0.029 (4)	0.023 (3)
O1WB	0.133 (8)	0.045 (4)	0.044 (4)	−0.006 (5)	−0.005 (5)	0.018 (3)

### Geometric parameters (Å, º)

**Table:**

Sn1—Cl2	2.4470 (10)	C3—C4	1.372 (15)
Sn1—Cl2^i^	2.4470 (10)	C3—C2	1.366 (15)
Sn1—Cl1B^i^	2.371 (6)	C3—C6	1.48 (2)
Sn1—Cl1B	2.371 (6)	C4—C5	1.354 (10)
Sn1—Cl3B^i^	2.451 (7)	C5—N1	1.339 (11)
Sn1—Cl3B	2.451 (7)	N1—C1	1.358 (12)
Sn1—Cl1A	2.475 (7)	C1—C2	1.374 (12)
Sn1—Cl1A^i^	2.475 (7)	C6—N2	1.306 (14)
Sn1—Cl3A^i^	2.402 (4)	C6—N3	1.226 (16)
Sn1—Cl3A	2.402 (4)		
			
Cl2—Sn1—Cl2^i^	180.0	Cl1B—Sn1—Cl3A^i^	78.11 (14)
Cl2—Sn1—Cl3B^i^	87.17 (16)	Cl3B—Sn1—Cl3B^i^	180.0
Cl2^i^—Sn1—Cl3B^i^	92.83 (16)	Cl3B—Sn1—Cl1A^i^	105.72 (16)
Cl2—Sn1—Cl3B	92.83 (16)	Cl3B^i^—Sn1—Cl1A^i^	74.28 (16)
Cl2^i^—Sn1—Cl3B	87.17 (16)	Cl3A—Sn1—Cl2	89.55 (8)
Cl2^i^—Sn1—Cl1A	90.98 (14)	Cl3A^i^—Sn1—Cl2	90.45 (8)
Cl2—Sn1—Cl1A	89.02 (14)	Cl3A—Sn1—Cl1A	88.16 (12)
Cl2—Sn1—Cl1A^i^	90.98 (14)	Cl3A^i^—Sn1—Cl1A	91.84 (12)
Cl2^i^—Sn1—Cl1A^i^	89.02 (14)	Cl3A^i^—Sn1—Cl3A	180.0
Cl1B—Sn1—Cl2^i^	89.79 (15)	C4—C3—C6	123.3 (12)
Cl1B—Sn1—Cl2	90.21 (15)	C2—C3—C4	119.7 (15)
Cl1B^i^—Sn1—Cl2	89.79 (15)	C2—C3—C6	117.0 (12)
Cl1B^i^—Sn1—Cl2^i^	90.21 (15)	C5—C4—C3	120.1 (10)
Cl1B^i^—Sn1—Cl1B	180.0	N1—C5—C4	120.0 (9)
Cl1B—Sn1—Cl3B	87.92 (17)	C5—N1—C1	121.3 (9)
Cl1B^i^—Sn1—Cl3B	92.08 (17)	N1—C1—C2	119.2 (10)
Cl1B^i^—Sn1—Cl3B^i^	87.92 (17)	C3—C2—C1	119.5 (11)
Cl1B—Sn1—Cl3B^i^	92.08 (17)	N2—C6—C3	116.5 (11)
Cl1B—Sn1—Cl1A^i^	166.23 (14)	N3—C6—C3	118.0 (11)
Cl1B^i^—Sn1—Cl1A^i^	13.77 (14)	N3—C6—N2	125.4 (11)
Cl1B^i^—Sn1—Cl3A^i^	101.89 (14)		
			
C5—N1—C1—C2	−6 (3)	C2—C3—C6—N2	−42 (2)
C1—N1—C5—C4	6 (2)	C2—C3—C6—N3	142.5 (16)
N1—C1—C2—C3	2 (3)	C4—C3—C6—N2	135.9 (17)
C1—C2—C3—C4	0 (3)	C4—C3—C6—N3	−40 (3)
C1—C2—C3—C6	178.1 (16)	C2—C3—C4—C5	0 (3)
C6—C3—C4—C5	−177.7 (14)	C3—C4—C5—N1	−3 (2)

Symmetry code: (i) −*x*, −*y*+1, −*z*+1.

### Hydrogen-bond geometry (Å, º)

**Table:**

*D*—H···*A*	*D*—H	H···*A*	*D*···*A*	*D*—H···*A*
N1—H1···O1*WA*^ii^	0.86	1.96	2.760 (15)	154
N1—H1···O1*WB*^ii^	0.86	1.87	2.649 (15)	149
N2—H2*A*···Cl2^i^	0.86	2.68	3.431 (11)	147
N3—H3*A*···O1*WA*^iii^	0.86	2.13	2.961 (16)	162
N3—H3*A*···O1*WB*^iii^	0.86	1.96	2.795 (16)	163
N3—H3*B*···Cl1*A*^iv^	0.86	2.69	3.093 (16)	110
N3—H3*B*···Cl3*B*^iv^	0.86	2.56	3.420 (16)	175
O1*WA*—H1*WA*···Cl2^v^	0.85	2.77	3.415 (8)	134
O1*WA*—H1*WB*···Cl3*A*^vi^	0.85	2.41	3.154 (9)	147
O1*WB*—H1*WC*···Cl1*A*^v^	0.85	2.60	3.305 (10)	142
O1*WB*—H1*WC*···Cl1*B*^v^	0.85	2.36	3.085 (10)	144
O1*WB*—H1*WC*···Cl1*A*^vii^	0.85	2.69	3.251 (12)	124
O1*WB*—H1*WC*···Cl1*B*^vii^	0.85	2.83	3.396 (13)	126
C1—H1*A*···Cl3*A*^viii^	0.93	2.67	3.561 (17)	161
C1—H1*A*···Cl3*B*^viii^	0.93	2.43	3.356 (17)	174
C5—H5···Cl1*A*^vii^	0.93	2.80	3.674 (12)	157
C5—H5···Cl1*B*^vii^	0.93	2.56	3.385 (12)	149

Symmetry codes: (i) −*x*, −*y*+1, −*z*+1; (ii) −*x*+1, −*y*+4, −*z*+2; (iii) *x*, *y*−1, *z*; (iv) −*x*+1, −*y*+2, −*z*+2; (v) *x*, *y*+1, *z*+1; (vi) *x*, *y*+2, *z*+1; (vii) −*x*+1, −*y*+3, −*z*+2; (viii) −*x*+1, −*y*+2, −*z*+1.
